# Dual-Task Balance Training for Motor Skill Development among Children with Intelligence Quotient Discrepancy

**DOI:** 10.1155/2022/2822171

**Published:** 2022-01-15

**Authors:** Ebrar Atak, Zeliha Candan Algun

**Affiliations:** Department of Physiotherapy and Rehabilitation, Institute of Health Sciences, Istanbul Medipol University, Istanbul, Turkey Zip Code 34810

## Abstract

The motor skills of people with mental disabilities are reportedly reduced compared with those of their peers. Therefore, any task incorporating both motor and cognitive skills was hypothesized to provide better motor recovery. The aim of this study is to find the effect of dual-task balance training (DTBT) on motor skill development in children of 6–13 years with intelligence quotient discrepancy (IQD) (score: 50–79). Overall, 30 individuals with mental disabilities aged 6–13 years having an IQ score of 50–79 were included. The participants were randomly divided into two groups that received dual-task training and standard balance training, respectively. IQ was measured with the Wechsler Intelligence Scale for Children-Revised, motor proficiency with the Bruininks–Oseretsky test, reaction time with COGNIBOARD, and balance with Functional Reach Test scores. Intervention was provided twice a week for 12 consecutive weeks. Participants in both groups showed higher test scores in all tests after the training program. Both training programs positively affected the motor performance of the participants. The DTBT was more effective in improving balance performance than the standard balance training. DTBT is a better tool than conventional balance training for improving motor skills and balance in children of 6–13 years with IQD (score: 50–79).

## 1. Introduction

Motor development is the process of generating, controlling, and using physical forces in bringing about movements that range from involuntary to voluntary. The uniqueness of any biological model is the adaptation, for which motor behavior is no exception. Adaptive control of movement depends on core psychological functions mainly perception and cognition, which are utilized for planning and guiding movements [1, 2]. Motor development is the transition from simple, unorganized, and unskilled movements to regular, complex, and purposeful movements [3]. The efficiency of cognitive and motor systems is reflected in both early rapid motor development and cognitive functions developed later throughout life [4].

Recent theories state that executing a new motor skill will result in a new cognitive process that forms more neural networks and motor programs for future tasks. This also enhances their perceptual skills by interacting with new objects and people in the environment. With adaptive motor behavior heavily depending on cognitive development, training motor skills among patients with cognitive deficits become a major challenge. Cognition is an umbrella term that pertains to many functions like memory, attention, comprehension, orientation, and language. One among them is intelligence, which integrates cognitive functions in the process of reasoning, problem-solving, and learning. One of the common IQ problems seen in school-going children is intelligence quotient discrepancy (IQD), which is defined as the difference between verbal IQ and performance IQ obtained from the Wechsler Intelligence Scale [5]. It is proven in the past that IQD coexists with neurological deficits ranging from simple reading disabilities and arithmetic disabilities to complex language, speech, or learning difficulties [6].

In children, motor skills and IQ have a strong relationship with executive functions. In preadolescents compared to postadolescents, there is a stronger correlation between motor and cognitive skills [7]. Yet there are no studies, to our knowledge, that exist to prove the relationship between training executive function and motor skill development among children with IQD. It is found that complex motor tasks that utilize conceptual problem-solving ability, mental efficiency, and language-related skills will be associated with IQ, whereas simple motor tasks and perceptual tasks were not [8]. In these lines, we found from literature that during dual-task activity, cognitive resources are essential in postural control like standing [9] and walking unlike in normal circumstances [10] among children and adolescents. This was the main gap identified in the literature, where there were no studies that trained the balance component that was compromised in children with IQD.

Dual-task balance training (DTBT) has been in use in the management of many neurological disorders of all ages [11]. DTBT not only integrates cognitive and motor skill components in the process but also helps the two components augment each other. Patients with IQD showed poor attention, which plays a vital role in the executive function, which can be well trained by dual-task performance. Although there is limited knowledge on attention in traditional balance training, attention has an important place in DTBT. The compelling effect of DTBT on attention and concentration suggests that it is more effective than traditional balance training in developing motor skills. This was the main reason behind selecting DTBT as an intervention tool amidst so many interventions that focus individually on each of the components. Hence, in this study, we tried to find the effect of DTBT on improving motor functions among children with IQD in the age range of 6–13 years. Further, this study was aimed at investigating the role and importance of adding a well-designed DTBT physiotherapy program to educate the IQD individuals on the development of motor skills. Improvement in motor skills can also support the IQD individuals to cope with the difficulties due to motor skill losses, which they encounter during their daily activities. The research question is unique because so far, there were no interventions available for the balance outcomes in IQD and further the utility of DTBT has not been tested in IQD to our knowledge. Moreover, the effectiveness of DTBT studies on preventing functional losses due to motor skill losses will increase the importance of physiotherapists in the rehabilitation and education of the IQD individuals.

## 2. Materials and Methods

### 2.1. Subjects

The comparative study was designed and conducted in Gerçek Dünya Special Education and Rehabilitation Center between 2018 and 2019. The results obtained in the study were archived by the authors within the scope of personal data protection. Thirty children between the ages of 6 and 13 years were selected who had IQ score that ranged between 50 and 79 assessed using the Wechsler Intelligence Scale for Children-Revised [12]. All the subjects had a health board report showing that they are mentally disabled, being able to follow instructions. Subjects who had a genetic or chronic disease or syndrome, having a disease of the musculoskeletal system, not being suitable for the methods to be used in evaluations, or not being able to complete the evaluations were excluded from the study.

### 2.2. Ethics Approval and Consent

Written informed consent was obtained from the parents or the caretakers after sufficient education about the research. This study was approved by the ethics committee of the Istanbul Medipol University Non-Interventional Clinical Studies (ethics committee approval number 10840098-604.01.01-E.53730 and approval date 12.21.2018) for noninvasive clinical studies and was conducted in accordance with the Declaration of Helsinki.

### 2.3. Intervention

The 30 subjects were randomized into two groups at a 1 : 1 ratio with 15 subjects in each group. A simple random sampling was used with a random number table method [13]. The experimental group received DTBT in the scope of cognitive rehabilitation whereas the control group received standard balance exercise training. The intervention was provided twice a week for 12 consecutive weeks.

The DTBT program is comprised of 10 min warm-up exercises where the first 5 min of the warm-up included walking on a treadmill and the next 5 min included walking to the front, side, and back on normal and soft ground, followed by a 20 min DTBT in two parts, each lasting 10 min. During the first 10 min of the exercise, a Stroop task was performed on the balance ball in the sitting position. The purpose of this task was to improve neurocognitive processes, such as focused attention, response inhibition, resistance to interfering effects, and information processing speed. In the second 10 min of the training program, the participant performed exercises in the multitask mode of the COGNIBOARD in a standing position on the Bosu balance ball. In this mode, a random light was switched on and off and the participant was asked to press the button that previously switched off after a second light switched on and then continue this process for 10 min. This mode targeted neurocognitive processes, such as attention, short-term memory, hand-eye coordination, focus, and perception [14–18].

The subjects in the control group also received warm-up exercises similar to the experimental group; subsequently, the participant was asked to perform balance exercises in two sets of 10 min in the presence of a physiotherapist. The first 10 min part of the exercises was designed to include the tasks of transferring weight to the front, back, right, and left, touching targets in different directions, catching a moving object, and balancing while a force is applied by the physiotherapist to disturb balance while the participant was in a sitting position on the balance ball. In the second 10-minute part, the same exercises were performed with the participant standing in an upright position on the Bosu balance ball [19–21].

### 2.4. Outcome Measures

Three standard outcome measures (SOM) used for the study were the Bruininks–Oseretsky Motor Proficiency Test (BOT), Functional Reach Test [22], and COGNİBOARD. Evaluations were performed by a specialist physiotherapist. The SOM was applied at the baseline, at the completion of the intervention period of 12 weeks, and after 3 months of completion of the intervention. Subjects were treated twice a week for 12 consecutive weeks, which made the participation of children highly convenient. In our clinical practice, we have observed that when children are asked to attend therapy sessions for longer time periods, such as 5 times a week, they often skip sessions as well as therapies. Therefore, it was decided to set 12 weeks as the optimum intervention period. To observe the long-term effects of the application, it was suggested to wait for the entire duration of the application, i.e., 3 months (12 weeks), and then terminate the application. BOT2 has been the most widely used standard measure that comprises goal-oriented activities designed to measure a wide range of motor skills in people between the ages of 4 and 21 years which takes 15–20 min to complete with the highest possible score of 88 [23, 24]. The Functional Reach Test [22] was used as a measure of balance outcome. The COGNIBOARD comprises 64 illuminated buttons placed on a panel that can be adjusted as per the individual's height. The arrangement of keys creates five specially designed rings. The circle's radius formed by the outermost ring is 55 cm. Audible warnings are used while the individual is performing the exercises as per light instructions. Thus, tactile, visual, proprioceptive, and auditory feedback is provided; it motivates children to exercise with its fun sound mode. COGNIBOARD includes eight increasingly difficult modes, each of which is designed for a different part of the body and in which the keys flash in various combinations. There are two modes available, namely, the reaction time mode and the multitask mode out of which the latter was used in the study. This mode enables the brain to perform multiple tasks in a versatile and simultaneous manner to stimulate synaptic connections and brain signals in the brain. COGNIBOARD can provide visual information, such as speed, accuracy, and productivity increase. Moreover, visual perception is improved in static and dynamic environments that can help compensate for visual impairment in motor, cognitive, and sensory impairments because of neurological injuries and diseases.

### 2.5. Procedure of Using COGNIBOARD for the Study

The participants were positioned on a standard Bosu balance ball with a diameter of 65 cm and a height of 25 cm. Subsequently, the height of the COGNIBOARD is adjusted such that the center of the COGNIBOARD lights is aligned with the participant's eyes. The distance between the COGNIBOARD and the person who will run the test is adjusted as per the person's height and arm length. When the person lifts their arm on the Bosu balance ball, the person is asked to touch the stimulating lights on the topmost and bottommost ring among the circularly placed stimulating lights on the COGNIBOARD without bending their knees and bending forward from the waist. The Bosu balance ball is positioned at a distance from which the person can touch the illuminated stimulating buttons on the topmost and bottommost rings. COGNIBOARD is adjusted based on the outermost ring lights that the person can reach before the test is performed.

### 2.6. Statistical Analysis

The SPSS 25.0 statistical analysis software was used for data analysis. The statistical analysis was performed at 95% confidence interval with a 0.05 significance level. Between-group analysis was performed using the Mann–Whitney *U* test, and the within-group analysis was done using the Friedman test. In case of significant difference over a different period in each group, a post hoc analysis will be done using the Bonferroni test.

## 3. Results

**Figure 1:**
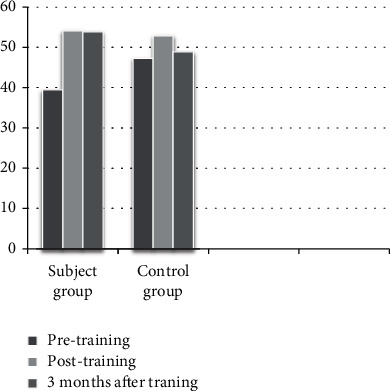
Distribution of the scores of the participants from the Bruininks–Oseretsky Motor Proficiency Test.

**Figure 2:**
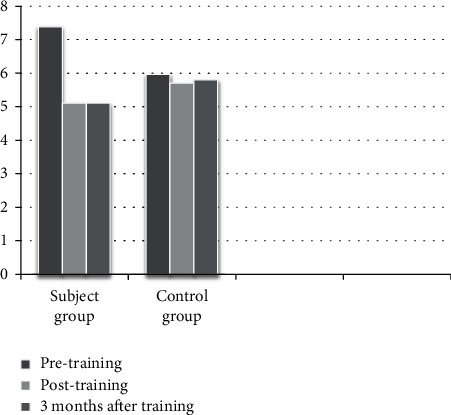
Distribution of scores of participants from the COGNIBOARD test.

**Figure 3:**
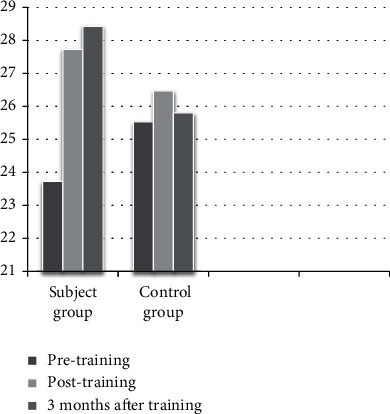
Distribution of participants' scores from the Functional Reach Test.

A total of 30 accounted for the final results of the study. Every group comprised 15 subjects completing the intervention and 3 months of follow-up. The characteristics of the participants of both groups are displayed in Table 1.  Table 2 and Figures 1–3 show both groups' performance in BOT2 and COGNİBOARD as well as Functional Reach Test in the outcome analysis at three given time points.

Between-group analysis of the BOT of motor proficiency scores shows that there was a significant difference at the baseline with the control group performing better than the experimental group (*Z* = −3.26 and *p* < 0.001), but subsequently, there was no difference between the groups in the posttest (*Z* = −0.41 and *p* = 0.67) and there was a significant difference thereafter at the end of the third month (*Z* = −2.18 and *p* = 0.02). In the COGNIBOARD scores, there was a significant difference in the pretest scores (*Z* = −2.38 and *p* = 0.017), but there was no significant difference following the intervention (posttest 1: *Z* = −0.97 and *p* = 0.32; posttest 2: *Z* = −1.30 and *p* = 0.19). In the Functional Reach Test, there was no significant difference between the pretest values (*Z* = −1.07 and *p* = 0.28), but there were a marginally significant difference between the groups at posttest 1 (*Z* = −2.01 and *p* = 0.04) and a highly significant difference between the posttest 2 values (*Z* = −3.40 and *p* < 0.001) with the experiment group performing better. In the within-group analysis, there was a significant difference in the analysis of variance on ranks; hence, post hoc analysis was done for all the three outcome measures in both the groups, which are displayed in Table 3.

## 4. Discussion

The study purpose was to find the effect of DTBT in the enhancement of motor performance compared to the conventional balance training program among children with IQD (50–79) at the age of 6–13 years. For this study dual-task activity, cognitive and motor tasks were provided. Different approaches have been proposed on how DTBT can improve cognitive and motor performance as it reduces attention requirements by focusing on automating an individual task [25]. The same has been used in the current study, which showed that there was a good improvement in motor performance and balance performance when DTBT was used compared to conventional balance training. These results were observed in the past in other neurological deficits as well [14, 26].

The children at the age of 6–13 had been performing poorly in terms of motor task but performed better cognitive task compared to the more aged children [27]. This was the main reason behind the selection of 6–13 years of age for this study. This also explains why a motor task and a cognitive task were both incorporated into the dual-task intervention. When these conditions are met, DTBT has both motor and cognitive benefits.

In this study, the motor function and balance performance were analyzed using three SOM. The balance performance, measured using the Functional Reach Test, has significantly improved with DTBT, even though there was no baseline homogeneity, with the control group showing better balance skills than the experimental group. There was a consistent improvement in the balance performance, and the benefits were at their maximum even after the intervention has seized for 3 months. Similarly, previous studies have demonstrated that gait and balance control are often improved using DTBT, and there is a broad consensus that it is necessary to increase attention capacity [28–30].

BOT2 motor proficiency scores and COGNIBOARD scores (reaction time) were used to assess the motor skills of the participants, which showed that DTBT has a positive effect on motor skills. The BOT2 scores were not equal at baseline with the control group performing better than the experimental group. The differences have evened out at the end of the 12 weeks, and the experimental group had shown a consistent improvement thereafter, which was not seen in the control group at the end of the 3rd month. This proves that DTBT helps in developing a better motor strategy and helps in motor learning that has resulted in a sustained improvement even after the intervention was stopped. There was a slight change in the trend in COGNIBOARD scores. Here, the difference existed in baseline similar to BOT2 and that was evened out at the end of 12 weeks, but thereafter both scores remained the same after the 3-month follow-up. In the within-group analysis, it is seen that both interventions have shown a significant improvement in all three outcome measures when the intervention was continuously provided till 12 weeks. However, the motor skills did not improve with control intervention during the follow-up, which emphasizes the role of motor learning that has happened with DTBT. There was no marked difference in the prognosis of balance function with both interventions as far as significance, but the presence of baseline difference has to be considered here.

These results may be due to the fact that performing a cognitive process during walking has a disruptive effect on gait, balance, and posture as these are complex physical activities. Although this disruptive effect differs by age groups, it is observed that cognitive performance is decreased during walking in both children and young adults. It is documented that there is a developmental trend in the sources of attention used to control gait in typical children [9, 31]. As it is understood, cognitive performance has an undeniable effect on balance and postural control even during basic physical activities such as walking.

Apart from motor performances, balance was taken up as an important outcome in this study because of its role in motor and cognitive development. It is claimed in the past that growth retardation negatively affects the motor learning process; it makes the automatization of motor movements difficult, which in turn leads to balance and coordination problems [32]. It has been suggested that an important cause of the motor retardation that takes place in growth retardation is attention deficit. It is known that, during dual-task functions, children with and without attention deficit experience performance losses. This once again justifies the benefits of the inclusion of a cognitive task as a part of DTBT.

It is reported that, among motor skills, the greatest loss occurs in coordination, balance, speed, strength, and manipulative skills [33]. Therefore, based on the past data in the literature and the data of our study, people with mental disabilities should receive both cognitive training and training that supports motor skills, such as balance training. Similar results have been obtained among the aged population, where DTBT had positive effects on motor function and balance function in elderly individuals, stroke survivors, and people with mental disabilities [34]. In the current study, there was a significant difference in the baseline data of the outcome measures, which clearly states that the control group was well placed in terms of motor performance and balance before the commencement of the intervention. This can be justified by the small sample selected for the study, which is a limitation of this study. The researchers also recommend for future study analyzing COGNIBOARD's usage as a tool in effectively and sensitively measuring motor skills. In this study, all the data were considered nonparametric as the sample size was small and was not normally distributed. It is also recommended to use some objective tools in measuring the changes in future studies, which will help strengthen our results.

## 5. Conclusions

The study concludes that though both conventional balance training and DTBT training were effective in improving the motor skills and balance performance among children of 6–13 years with IQD, DTBT showed a better improvement, which sustained even after the intervention was stopped. This study recommends future studies to explore the research question with more sample size and also in other cognitive deficits apart from IQD among various age groups.

## Figures and Tables

**Table 1:** Characteristics of the individuals who participated in the study.

	Experimental group		Control group	
	Mean	SD	Mean	SD
Weight	27.80	4.72	30.33	8.91
Size	130.87	6.57	128.47	6.91
BMI	16.17	1.85	18.09	3.32
Age	8.87	1.45	8.20	1.52
Intelligence score (WISC-R)	69.00	2.97	67.87	4.62

SD: standard deviation; BMI: body mass index; WISC-R: Wechsler Intelligence Scale for Children-Revised.

**Table 2:** Performance of both groups in the BOT2, COGNİBOARD, and Functional Reach Test in the outcome analysis at three given time points.

Outcome measure	Group	Pretest (mean ± SD)	After 12 weeks (mean ± SD)	After 3 months (mean ± SD)
BOT	Experimental	39.40 ± 5.22	54.07 ± 5.00	53.80 ± 5.28
	Control group	47.27 ± 6.98	52.87 ± 6.70	48.87 ± 6.37
COGNIBOARD	Experimental	7.38 ± 1.41	5.11 ± 1.19	5.11 ± 1.19
	Control group	5.97 ± 1.51	5.70 ± 1.61	5.80 ± 1.55
FRT scores	Experimental	23.73 ± 2.21	27.73 ± 1.87	28.43 ± 2.20
	Control group	24.53 ± 1.76	26.47 ± 1.18	25.80 ± 1.37

BOT: Bruininks–Oseretsky Motor Proficiency Test; FRT: Functional Reach Test; SD: standard deviation.

**Table 3:** Summary of results of post hoc analysis of all the three outcomes in both the groups.

Experimental group comparison (*p* value)	BOT2	COGNIBOARD	FRT
Pre- vs. posttest 2	0.001	0.85	0.001
Pre- vs. posttest 1	0.001	0.001	0.001
Posttest1 vs. 2	0.36	0.001	0.14
Control group comparison (*p* value)	BOT2	COGNIBOARD	FRT
Pre- vs. posttest 2	0.06	0.70	0.003
Pre- vs. posttest 1	0.001	0.001	0.001
Posttest1 vs. 2	0.001	0.03	0.12

BOT: Bruininks–Oseretsky Motor Proficiency Test; FRT: Functional Reach Test.

## Data Availability

The data used to support the findings of this study are included within the article.
